# Activation of Cannabinoid Receptor 1 in GABAergic Neurons in the Rostral Anterior Insular Cortex Contributes to the Analgesia Following Common Peroneal Nerve Ligation

**DOI:** 10.1007/s12264-023-01029-6

**Published:** 2023-02-11

**Authors:** Ming Zhang, Cong Li, Qian Xue, Chang-Bo Lu, Huan Zhao, Fan-Cheng Meng, Ying Zhang, Sheng-Xi Wu, Yan Zhang, Hui Xu

**Affiliations:** 1 Department of Neurobiology and Collaborative Innovation Center for Brain Science, School of Basic Medicine, The Fourth Military Medical University, Xi’an, 710032 China; 2 Department of Anesthesiology, Heze Municipal Hospital, Heze, 274031 China; 3 Department of Cardiovascular Surgery, Xi’an International Medical Center Hospital, Xi’an, 710100 China; 4 Department of Basic Medical Laboratory, The General Hospital of Western Theater Command, Chengdu, 610083 China

**Keywords:** Rostral agranular insular cortex, Cannabinoid receptor 1, Neuropathic pain, Dorsolateral fasciculus, GABAergic neuron

## Abstract

The rostral agranular insular cortex (RAIC) has been associated with pain modulation. Although the endogenous cannabinoid system (eCB) has been shown to regulate chronic pain, the roles of eCBs in the RAIC remain elusive under the neuropathic pain state. Neuropathic pain was induced in C57BL/6 mice by common peroneal nerve (CPN) ligation. The roles of the eCB were tested in the RAIC of ligated CPN C57BL/6J mice, glutamatergic, or GABAergic neuron cannabinoid receptor 1 (CB1R) knockdown mice with the whole-cell patch-clamp and pain behavioral methods. The E/I ratio (amplitude ratio between mEPSCs and mIPSCs) was significantly increased in layer V pyramidal neurons of the RAIC in CPN-ligated mice. Depolarization-induced suppression of inhibition but not depolarization-induced suppression of excitation in RAIC layer V pyramidal neurons were significantly increased in CPN-ligated mice. The analgesic effect of ACEA (a CB1R agonist) was alleviated along with bilateral dorsolateral funiculus lesions, with the administration of AM251 (a CB1R antagonist), and in CB1R knockdown mice in GABAergic neurons, but not glutamatergic neurons of the RAIC. Our results suggest that CB1R activation reinforces the function of the descending pain inhibitory pathway *via* reducing the inhibition of glutamatergic layer V neurons by GABAergic neurons in the RAIC to induce an analgesic effect in neuropathic pain.

**Supplementary Information:**

The online version contains supplementary material available at 10.1007/s12264-023-01029-6.

## Introduction

Neuroimaging studies have shown that increases in glutamate and decreases in gamma-aminobutyric acid (GABA) are responsible for cortical hyperactivity and hyperalgesia/allodynia in chronic pain patients. The rostral agranular insular cortex (RAIC) is associated with physiological pain and interacts with areas involved in cognitive and emotional control [1, 2]. Our previous study showed that the enhancement of excitatory synaptic transmission in the insular cortex (IC) contributes to neuropathic pain [3, 4]. Moreover, the excitatory neurotransmitters glutamate and aspartate were increased, but there were no significant changes in GABA levels of the IC in rats with chronic constriction injury of the sciatic nerve [5]. These studies suggest that an imbalance between excitatory and inhibitory synaptic transmission in the RAIC might contribute to hyperalgesia. However, the possible cellular and molecular mechanisms underlying this imbalance in the RAIC remain unclear under the neuropathic pain state.

Both depolarization-induced suppression of inhibition (DSI) and excitation (DSE) are forms of short-term plasticity, which modulate neurotransmitter release by inhibiting the excitability of either presynaptic interneurons or excitatory neurons [6, 7]. They are also produced in the postsynaptic neuron in the “on-demand” form. An elevation of postsynaptic Ca^2+^ concentration induced by depolarization is the trigger for DSI/DSE [8]. Then, the release of endocannabinoids and consequent activation of presynaptic cannabinoid receptor type 1 receptors (CB1Rs) result in DSI or DSE [9]. Many studies have reported that DSI or DSE is widely expressed in pyramidal neurons and regulates the excitability of postsynaptic neurons in the cerebral cortex in opposite directions [10]. DSI and DSE have been recorded in pain-related cortices such as the anterior cingulate cortex (ACC) and the medial prefrontal cortex (mPFC) [11, 12]. Whether DSI and DSE occur in the RAIC is still unclear. In the inflammatory pain model, DSE amplitude is decreased in the ACC [12]. DSI is absent in the mPFC of an arthritis pain model [11]. These findings indicate that the changes in DSE and DSI in the cortices contribute to chronic pain. Whether DSI and DSE undergo changes in the RAIC under neuropathic pain remains unknown.

The endocannabinoid (EC) system is involved in hyperalgesia or analgesia [3, 13–15]. It has been reported that CB1Rs are significantly increased in the IC of rats with neuropathic pain [14, 16]. The activation of CB1Rs in the IC decreases the nociceptive behaviors of rats with sciatic nerve ligation [17]. A potent CB1R agonist HU210 induces analgesia in GABAergic neurons of the prefrontal ventrolateral orbital cortex of mice with common peroneal nerve (CPN) ligation [18]. The activation of CB1Rs decreases the GABA current but not the excitatory current in cortical neurons of normal mice, which increases pyramidal neuron activity [19, 20]. The activation of CB1Rs in GABAergic or glutamatergic neurons might account for the different effects in the neuropathic pain state. Furthermore, neural tracing and physiological methods have shown that the RAIC participates in pain modulation *via* the descending pain inhibitory system [1]. The analgesic effect of cannabinoids is blocked after a dorsolateral funiculus (DLF) lesion [21–24]. However, whether the activation of CB1Rs in the RAIC modulates chronic pain *via* the DLF is unknown.

In the present study, we examined the balance between the excitatory and inhibitory synaptic transmission of RAIC layer V neurons following CPN ligation, the change of DSI, the effect of activation of CB1Rs in either glutamatergic or GABAergic neurons of the RAIC, and whether the effect of the activation of CB1Rs in the RAIC modulates neuropathic pain *via* the DLF.

## Materials and Methods

### Animals

The protocol of this study was approved by the Laboratory Animal Welfare and Ethics Committee of the Fourth Military Medical University (IACUC-20130901). All animals were housed and cared for in line with the guidelines set forth by the International Association for the Study of Pain. Adult wild-type C57BL/6J male mice were purchased from the Laboratory Animal Center of the Fourth Military Medical University (Xi’an, Shaanxi Province, China). CB1R-floxed male mice on a C57BL/6J background were used (generous gifts from Prof. Wei Ren, Key Laboratory of Modern Teaching Technology, Ministry of Education, Shaanxi Normal University, Shaanxi Province, China). Adult male mice (a total of 195 mice) were randomly divided into groups. A randomized, double-blind controlled experiment was conducted in pain behavior experiments. Preparation of the pain model and pain behavior testing was performed between 08:00 and 12:00. Electrophysiological experiments were performed between 08:00 and 18:00. The animals were housed in a temperature- and humidity-controlled room with a 12-h light/dark cycle and had access to food and water *ad libitum*.

## CPN Ligated Mouse Model

Adult male mice were anesthetized with 1% pentobarbital sodium (0.1 mL/10 g, i.p.). Surgical procedures were performed as previously described [3]. The left leg was shaved (1 × 1 cm^2^) and the skin was cleaned with iodine. Surgical procedures commenced when reflexes to painful stimuli disappeared. An outstanding depression was visible between the anterior and posterior muscle groups when the left leg was lifted gently. Then, the skin was cut along this depression (1 cm). The CPN was visible under the posterior group of muscles. The CPN was separated with a glass needle and ligated only once with mouse tendon filaments (5–0). The ligation process was slow without an increased tightening of the knot. Finally, the skin was sutured using 5–0 silk and cleaned with iodine. In the sham group, the CPN was exposed but without ligation.

## Mechanical Pain Behaviors

Before testing, adult male mice (a total of 195 mice) were allowed to adjust for 30 min in an acrylic box (20 cm in length on a side). Responsiveness of the middle dorsum in hind paws to innocuous mechanical stimuli produced by a von Frey filament (0.008 g) was used to assess mechanical allodynia. The testing process was repeated 5 times in one session. The positive response rate was the percentage of positive responses in one session. To measure the bilateral paw withdrawal thresholds, a series of von Frey filaments from 0.008 g were applied to the middle dorsum of the hind paw in ascending order. As previously described, flicking, withdrawal, or licking was treated as a positive nociceptive response. Testing with each filament was repeated five times, and the paw withdrawal threshold (PWT) was defined as the minimal force of filaments inducing three or more positive responses.

## Cannula Implantation and Microinjection into the RAIC

Adult male mice were anesthetized with 1% pentobarbital sodium (0.1 mL/10 g, i.p.). The mouse head was secured on a stereotaxic frame, then a 24 gauge guide cannula was implanted bilaterally into the RAIC (1.5 mm anterior to bregma, 3.5 mm lateral from the midline, 4.0 mm beneath the surface of the skull). Mice were allowed to recover for one week after the cannula implantation. In the process of microinjection, mice were anesthetized with 2% isoflurane. Thinner 30-G cannulas were lowered into the RAIC (4.5 mm). A Hamilton syringe (10 μL) was fixed to a micro-injection pump and a needle was connected to the 30-G cannulas by a thin polyethylene tube. The CB1R agonist ACEA (2 μmol/L, 0.5 μL) and the CB1R antagonist AM251 (2 μmol/L, 0.5 μL) were infused into both sides of the RAIC at a rate of 0.5 μL/min. The microinjection needle was left in the injected site for at least 2 min after injection. At the end of the experiment, the injection site was confirmed by haematoxylin/eosin dye. The effect of ACEA on pain behaviors was not tested until the mice were awake and freely moving. The effect of an intervention on mechanical allodynia and PWTs was accessed within 30 min after injection.

## Dorsolateral Funiculus Lesion

Adult male mice were anesthetized with pentobarbital sodium (1%, 0.1 mL/10 g, i.p., a total of 41 mice). The skin over T3–T4 was cut along the midline. After laminectomy, the dura mater was pierced with a 26 gauge needle. The spinal cord was exposed carefully under a dissecting microscope. Then the mouse was fixed on the stereotaxic instrument to make sure the segment of the spinal cords was in a straight line. The bilateral dorsolateral funiculus (DLF) was damaged by a modified 25-gauge bevel-tipped needle (1.0 mm lateral from the midline, 1.0 mm beneath the surface of the dura mater) [25, 26]. A nearby autologous fat graft was placed over the laminectomy site. Muscles and skin were sutured using 5–0 silk. To avoid infection, 0.9% saline (37°C, 1 mL) and penicillin sodium (80 mg/kg, i.p.) were injected. In the sham group, the procedure was the same, but without injury to the DLF.

## Brain Slice Preparation and Electrophysiology

Adult male mice (a total of 63 mice) were anesthetized with 2% isoflurane. Based on the atlas of Franklin and Paxinos, slices (300 μm) through the RAIC were cut on a vibratome (VT1200S, Leica) in oxygenated artificial cerebrospinal fluid (ACSF) containing (in mmol/L): NaCl 124, KCl 2.5, MgSO_4_ 2, CaCl_2_ 2, NaHCO_3_ 25, NaH_2_PO_4_ 1, glucose 37, pH 7.4. The RAIC slices were transferred to a chamber with oxygenated ACSF at room temperature. Whole-cell electrophysiological recordings were made on the stage of a microscope (BX51W1, Olympus). Both miniature excitatory postsynaptic currents (mEPSC) and miniature inhibitory postsynaptic currents (mIPSCs) were recorded from layer V neurons in the RAIC with an Axon 200B amplifier (Molecular Devices). The recording pipettes (2–6 MΩ) were filled with intracellular fluid containing (in mmol/L): Cs-MeS_2_O_3_ 140, MgSO_4_ 1, NaCl 5, EGTA 0.5, HEPES 10, Mg-ATP 2, Na_3_-ATP 0.1, QX-314 2, Spermine 0.1, Phosphoicreatine 10. For mEPSC and mIPSC recordings, pyramidal neurons were held at − 60 mV and + 10 mV, respectively, for 5 min. The E/I ratio is the average amplitude ratio between the mEPSCs and mIPSCs [27]. EPSCs were recorded from layer V neuron with stimulation by a bipolar tungsten stimulating electrode that was placed in layers II/III of the RAIC. Picrotoxin (100 μmol/L) was added to the perfusate to block GABA_A_ receptor-mediated inhibitory synaptic currents. For the recording of IPSCs, CNQX (10 μmol/L) and APV (50 μmol/L) were added. The recording pipettes were filled with intracellular fluid containing (in mmol/L): CsCl 140, MgCl_2_ 1, NaCl 5, EGTA 0.5, HEPES 10, Mg-ATP 2, Na_3_-ATP 0.1, QX-314 2, Phosphoicreatine 10. To induce DSE and DSI, recorded neurons were held at –60 mV, and depolarizing stimuli were delivered (− 60 mV to 0 mV, duration 1 s, 5 s, 10 s). 30 sweeps were recorded at 0.2 Hz. The magnitudes of DSE and DSI were calculated as the percentage difference between the mean amplitude of three consecutive EPSCs or IPSCs after depolarization relative to the mean amplitude of 1 min of consecutive EPSCs or IPSCs before depolarization [28].

## Virus Injection

CB1R knockdown in glutamatergic neurons or GABAergic neurons in the RAIC was produced by the microinjection of the Cre viruses into Cnr1-floxed mice (a total of 49 mice). Adult male mice were anesthetized by pentobarbital sodium (1%, 0.1 mL/10 g, i.p.) and fixed in a stereotactic head frame. Cre virus (total 200 nL) pAAV2-mCaMKIIα-H2B-eGFP-P2A-cre-WPRE-pA or pAAV-GAD67-EGFP-2A-cre virus was microinjected into the bilateral RAIC at 20 nL/min (1.5 mm anterior to bregma, 3.5 mm lateral from the midline, 4.0 mm beneath the surface of the skull) in CB1R-floxed mice. A sharp glass pipette (diameter 30–35 μm) was left in the injection site for at least 10 min after injection. Finally, the wound was covered with sterile bone wax, and the skin was sutured with 5–0 silk. Three weeks later, CB1Rs were specifically knocked out in glutamatergic neurons with the administration of pAAV2-mCaMKIIα-H2B-eGFP-P2A-cre-WPRE-pA into the RAIC (Glu-CB1R-KO, 24 mice). And CB1Rs were knocked out in GABAergic neurons with the administration of pAOV-GAD67-EGFP-2A-cre (GABA-CB1R-KO, 25 mice). Then CPN-ligated mice were prepared for CB1R knockout in glutamatergic or GABAergic neurons.

## Histology

At the end of the pain behavior testing, mice were anesthetized with pentobarbital sodium (1%, 0.1 mL/10 g, i.p.). Phosphate-buffered saline (0.1 mol/L, 25 mL) and paraformaldehyde (4%, 25 mL) were perfused through the cardiovascular system. The brain and spinal cord were carefully isolated, removed, and stored in a sucrose solution (25%) for dehydration. The brain and spinal cord samples were serially sectioned coronally at 8–10 μm throughout the microinjection site and the lesion site. Slides were stained with Harris' hematoxylin and eosin and examined under a light microscope (VS200, Olympus).

## Statistical Analysis

All data are expressed as the mean ± SEM and were analyzed using GraphPad Prism 9.0 software (GraphPad Software, San Diego, CA). The normal distribution of data was verified by the D’Agostino and Pearson normality test. If data were normally distributed, parametric analyses were used. Changes in pain behaviors at various time points were assessed using a two-way repeated-measures analysis of variance (ANOVA) followed by the Bonferroni *post hoc* test for pairwise multiple comparisons. Differences in the expression of CB1R protein, the amplitude and frequency of mEPSCs and mIPSCs, the amplitude of the E/I ratio, and DSI and DSE in the two groups were tested using the unpaired *t*-test. The effects of ACEA on pain behaviors were analyzed using one-way ANOVA, with the Bonferroni *post hoc* test. One-way ANOVA was also applied to test the effect of DLF lesions on ACEA-induced analgesia. In all analyses, the level of significance was set at *P* <0.05.

## Results

### The E/I Ratio is Enhanced in Layer V Pyramidal Neurons of the RAIC After CPN Ligation

**Fig. 1:**
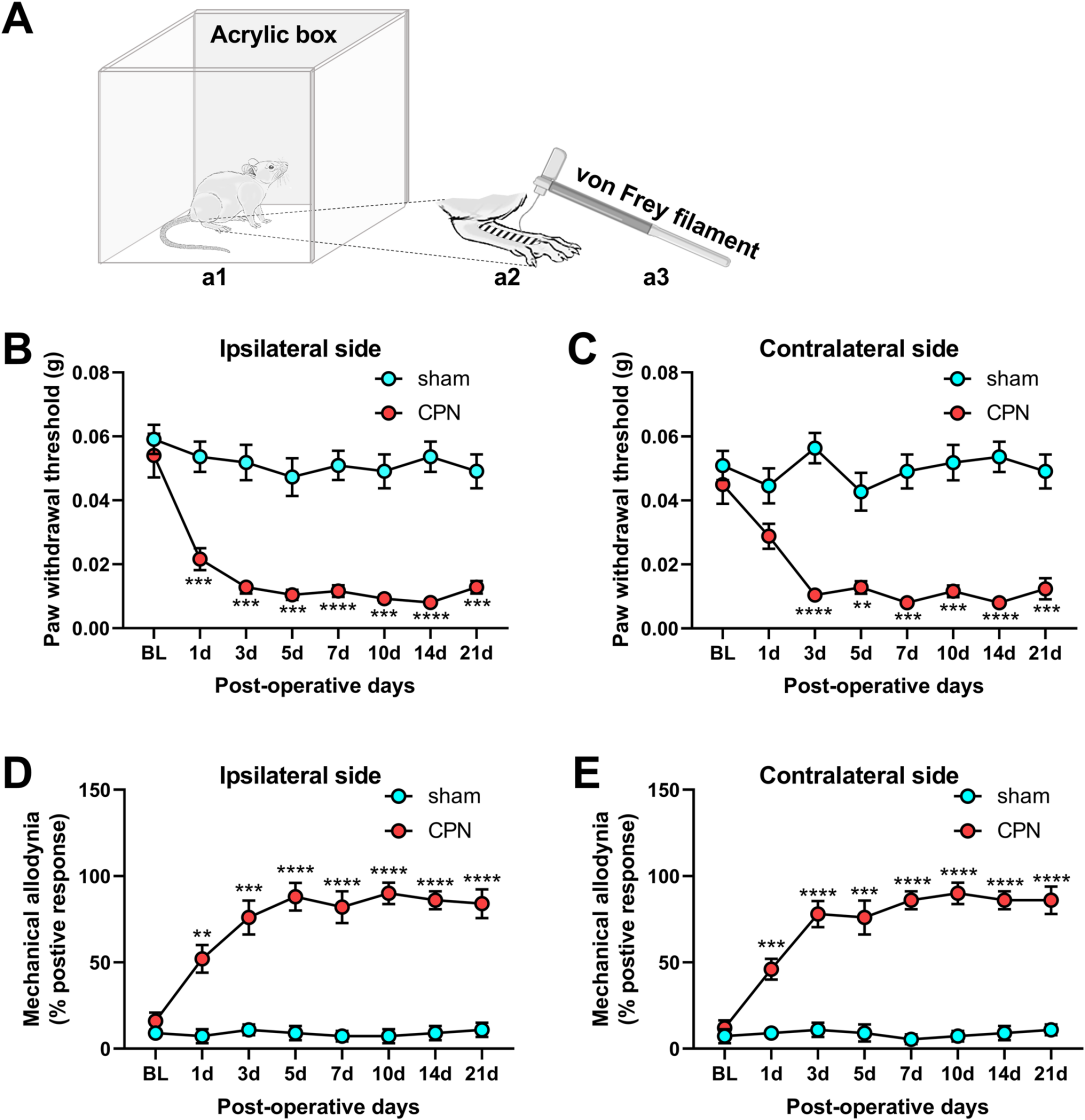
Bilateral mechanical allodynia and hyperalgesia in CPN mice. **A** Schematic of equipment for pain behavior testing, (**a1**) one mouse is placed in the square acrylic box (side length: 20 cm); (**a2**) the innervation area of the CPN (black lines); (**a3**) a von Frey filament is used for PWT testing. Bilateral mechanical hyperalgesia based on PWTs is measured on both sides. **B, C** There is a significant difference between the sham and CPN groups in the bilateral PWTs from day 1 to the end of the experiment. **D, E** Mechanical allodynia based on positive responses to innocuous stimuli (0.008 g von Frey filament) is used to evaluate the neuropathic pain of mice after CPN ligation. Compared to the sham group, the positive percentage of bilateral paw responses increase markedly after CPN ligation. Compared to the baseline values, **P <0.01, ***P <0.001, ****P <0.0001, two-way repeated-measures ANOVA followed by the Bonferroni *post hoc* test; sham group, 11 mice; CPN ligated group, 10 mice.

**Fig. 2:**
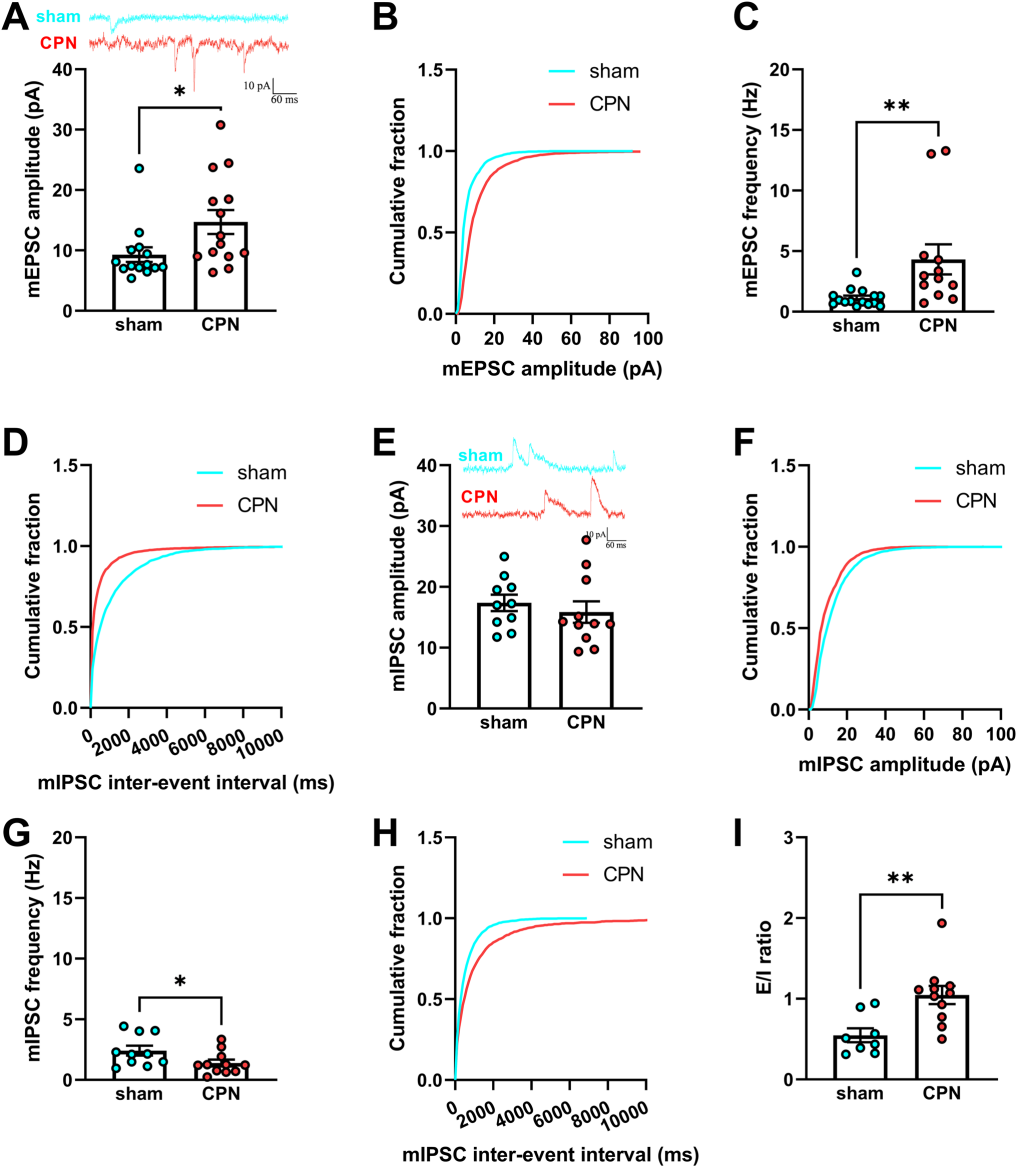
Increased E/I ratios of layer V pyramidal neurons in the RAIC following CPN ligation. **A** Representative mEPSCs were recorded in RAIC pyramidal neurons of sham mice (cyan) and mice following CPN ligation (red). The amplitude of mEPSCs in the RAIC of CPN-ligated mice is increased significantly (*P* = 0.0289). **B** The cumulative fraction of mEPSC amplitude in the RAIC of CPN-ligated mice is shifted to the right by a large margin (sham group, *n* = 15, 7 mice; CPN group, *n* = 14, 10 mice). **C** Marked difference in the mEPSC frequency in the RAIC of the two groups. **D** The cumulative fraction of mEPSC frequency in the RAIC of CPN-ligated mice is greatly shifted to the left (**A–D**, sham group, *n* = 15, 8 mice; CPN group, *n* = 12, 10 mice). **E** Representative mIPSCs recorded in RAIC pyramidal neurons of sham mice (cyan) and mice following CPN ligation (red). Compared to the sham group, the mIPSC amplitude in the RAIC is not changed statistically in the CPN-ligated group. **F** Changes of the cumulative fraction of mIPSC amplitude in the RAIC of CPN-ligated mice. **G** The frequency of mIPSCs in the RAIC of CPN-ligated mice decreases significantly. **H** The cumulative fraction of mIPSC frequency in the RAIC of CPN-ligated mice is right-shifted (**E–H**, sham group, *n* = 10, 7 mice; CPN group, *n* = 11, 8 mice). **I** E/I ratio (dividing the amplitude of mEPSCs into mIPSCs). There is a statistical difference in the E/I ratio between the two groups (sham group, *n* = 10, 7 mice; CPN group, *n* = 11, 8 mice). *P <0.05, **P <0.01, unpaired *t*-test for sham group *vs* CPN-ligated groups.

In the CPN-ligated mice, the bilateral PWTs were lower from day 1 to the end of the experiment (ipsilateral: *F*_19, 133_ = 1.988, *P <*0.05; and a significant group × time interaction *F*_7, 133_ = 4.659, *P <*0.001; contralateral: *F*_19, 133_ = 3.302, *P <*0.0001; Fig. 1B, C). And the percentage of positive responses of the bilateral paws increased markedly after CPN ligation (ipsilateral: *F*_19, 133_ = 1.797, *P <*0.05; and a significant group×time interaction *F*_7, 133_ = 10.45, *P* <0.0001; contralateral: *F*_19, 133_ = 3.302, *P* <0.0001; Fig. 1D, E). Furthermore, there was no statistical difference in behavioral hyperalgesia and allodynia between the bilateral paws in CPN-ligated mice (bilateral PWT hyperalgesia: *F*_7, 126_ = 1.472, *P =* 0.18; bilateral mechanical allodynia: *F*_7, 126_ = 0.302, *P =* 0.95; Fig. S1). The apparent pain hyperalgesia and allodynia of CPN-ligated mice provided an ideal neuropathic pain behavior for this study. Many studies have established that excitatory and inhibitory changes both take part in neuropathic pain. Compared to the sham group, both the amplitude and frequency of mEPSCs were significantly increased (amplitude, *P* = 0.0289; frequency, *P* = 0.009; Fig. 2A, C). At the same time, the cumulative fraction of mEPSC amplitude was shifted to the right, and the cumulative fraction of mEPSC frequency was shifted to the left (Fig. 2B, D). Although mIPSC amplitude tended to be decreased in CPN-ligated mice, there was no significant difference between the two groups (*P* = 0.5066, Fig. 2E). The mIPSC cumulative amplitude curve of CPN mice was shifted to the left to a small extent (Fig. 2F). The mIPSC frequency was markedly decreased and the cumulative curve was shifted to the right (mIPSCs frequency, *P* = 0.0495, Fig. 2H). To explore the integral changes in excitability (E) and inhibition (I), the E/I ratio of RAIC layer V pyramidal neurons was calculated for CPN-ligated mice. This E/I ratio was significantly enhanced in CPN-ligated mice (*P* = 0.0043, Fig. 2I).

## DSI Magnitude in Layer V Pyramidal Neurons of the RAIC is Increased After CPN Ligation

**Fig. 3:**
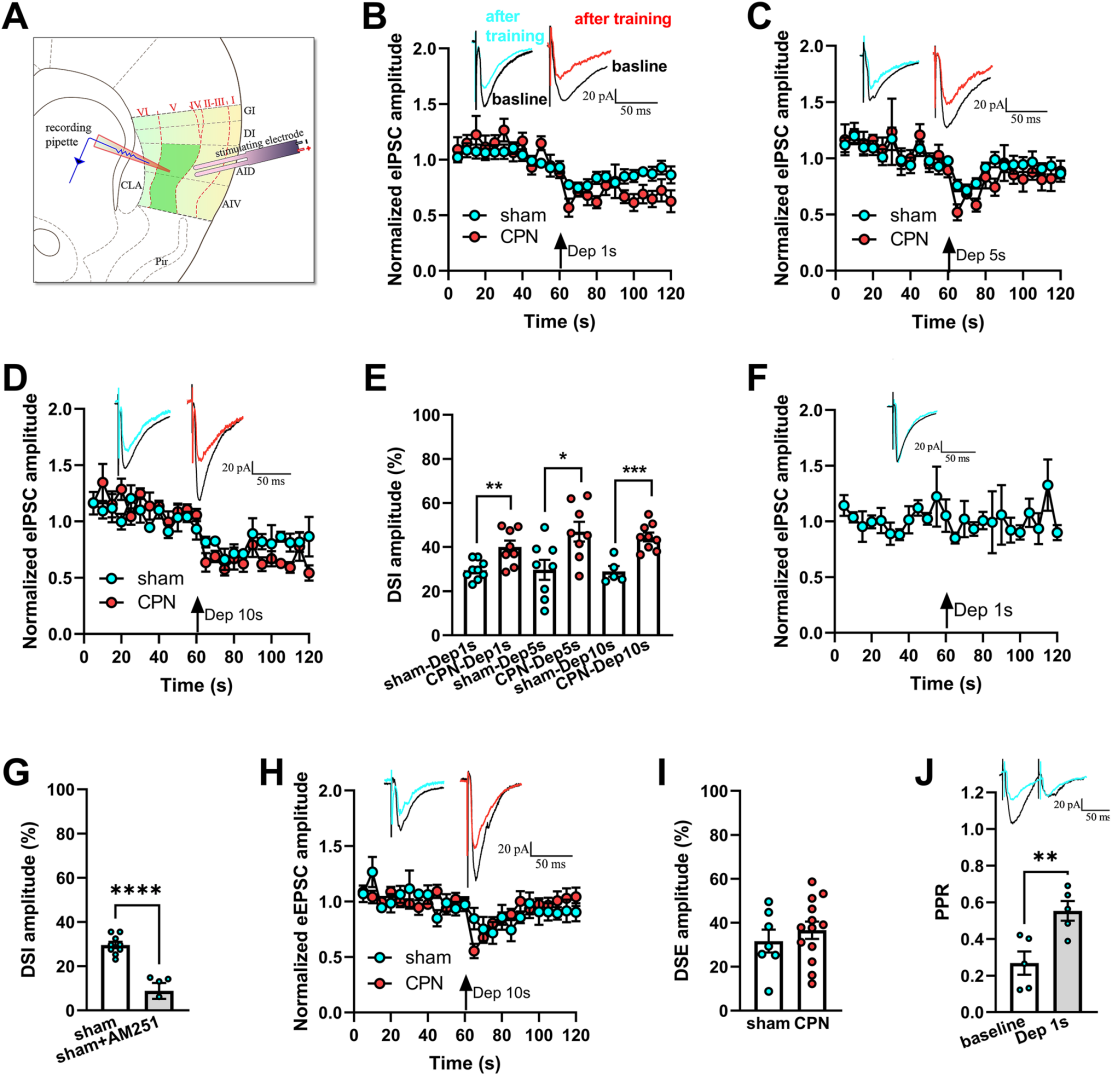
Increased DSI magnitude in layer V pyramidal neurons in the RAIC following CPN ligation. **A** Schematic of layer V neurons in the RAIC. In the electrophysiological study, the stimulating electrode was fixed in layers II–III. Pyramidal neurons in the RAIC layer V were recorded (green patch). AID, agranular insular cortex, dorsal part; AIV, agranular insular cortex, ventral part; CLA, claustrum; DI, dysgranular insular cortex; GI, granular insular cortex. **B–D** Protocol for different durations of depolarization (from –60 mV to 0) in layer V pyramidal neurons. Upper panels: representative currents under the given protocol. The baseline trace is the average before training (black). The post-training trace is the average of the next three sweeps after the protocol (cyan or red). The depolarization duration was 1 s (**B**, sham *n* = 14, 5 mice; CPN *n* = 7, 4 mice), 5 s (**C**, sham *n* = 8, 3 mice; CPN *n* = 8, 5 mice), 10 s (**D**, sham* n* = 7, 4 mice; CPN *n* = 8, 4 mice). The evoked IPSC amplitude decreases significantly after training. **E** DSI amplitude, the inhibitory rate of IPSCs was calculated at different durations. **F** Representative eIPSC currents recorded in RAIC pyramidal neurons of sham mice (black trace: baseline; cyan trace: after training). AM251 (2 μmol/L) is perfused in the recording fluid for 20 min before DSI recording. The inhibitory effect of the depolarization protocol on evoked IPSC amplitude disappeared (*n* = 5, 3 mice). **G** DSI is blocked by AM251. **H** Evoked EPSC amplitude is decreased when the depolarized protocol is applied (sham *n* = 7, 4 mice; CPN *n* = 15, 3 mice). Representative traces of eEPSCs from the sham and CPN ligation mice are shown above. **I** There is no significant difference in DSE amplitude in the two groups. **J** Representative traces with an interval of 50 ms recorded in layer V of the RAIC (black trace: baseline; green trace: depolarization for 1 s). The depolarization protocol (duration 1 s) amplified the PPR (*n* = 4, 3 mice). *P <0.05, **P <0.01, ***P <0.001, ****P <0.0001, compared to the sham group or baseline values; unpaired *t*-test.

To further identify the possible candidate for the changes in E/I ratio in RAIC layer V pyramidal neurons, we investigated DSI and DSE mediated by CB1Rs in these neurons. The evoked IPSC amplitude in pyramidal neurons was markedly decreased in both sham and CPN groups after the training protocol of DSI (from –60 mV to 0). The amplitude of DSI under different durations of depolarization was significantly increased in the CPN-ligated group (Fig. 3B: depolarization for 1 s, from 28.56% ± 4.35% to 40.21% ± 3.32%, *P* = 0.0045; Fig. 3C: depolarization for 5 s, from 23.40% ± 3.65% to 45.23% ± 5.10%, *P* = 0.0173; Fig. 3D: depolarization for 10 s, from 26.00% ± 1.97% to 49.86% ± 4.36%, *P* = 0.0004). As the depolarization time increased, the DSI amplitude of RAIC layer V pyramidal neurons from the CPN model increased markedly, but not in the sham group (Fig. 3E). The DSI was blocked by AM251 (DSI amplitude = 8.82 ± 3.61%, Fig. 3F; compared to the control, *P* <0.0001, Fig. 3G).

In addition, DSE was recorded in the layer V pyramidal neurons with a 10-s depolarization. The amplitudes of evoked EPSCs in the CPN and sham groups were both inhibited (Fig. 3H). However, there was no significant difference in DSE amplitude between the two groups (*P* = 0.5629; Fig. 3I). Both DSI and DSE existed in layer V pyramidal neurons of the RAIC in both sham and CPN mice. At the same time, to a certain extent, a presynaptic mechanism induced these changes in IPSCs and EPSCs. Taking the effect of depolarization for 1 s on IPSCs as an example, the PPR was significantly enhanced in layer V pyramidal neurons of the RAIC (*P* = 0.0092; Fig.3J). The increased PPR suggested that a presynaptic molecular mechanism mediates DSI or DSE.

## Analgesia is due to the Activation of CB1Rs in the RAIC in the Neuropathic Pain State

**Fig. 4:**
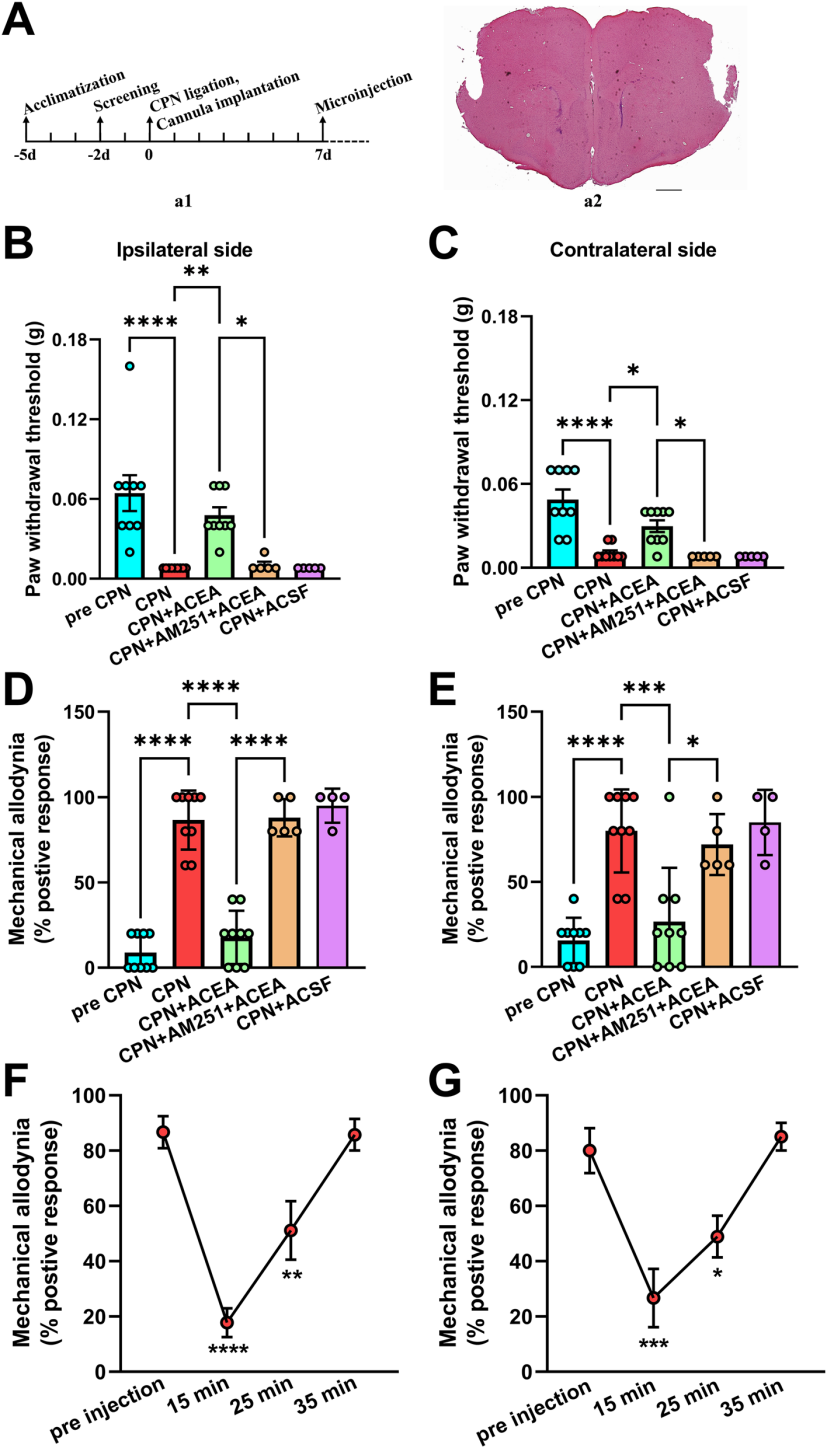
Inhibition of mechanical allodynia and hyperalgesia *via* CB1R activation in the RAIC following CPN ligation. **A** (**a1**) time course of behavioral experiments including the acclimatization, screening, CPN ligation, cannula implantation, and microinjection; (**a2**) a hematoxylin/eosin-stained section showing the cannula locations for microinjection (scale bar, 200 μm). **B, C** Bilateral PWTs increase significantly after ACEA (2 μmol/L, 0.5 μL) is microinjected into the RAIC (CPN 6 days *vs* ACEA, 9 mice). AM251 (2 μmol/L, 0.5 μL, 5 mice), a CB1R antagonist, is microinjected before ACEA (10 min). The analgesic effect of CB1R activation on the PWTs on both sides is blocked by AM251 (AM251+ACEA *vs* ACEA). **D, E** Mechanical allodynia of both sides is significantly inhibited by ACEA (CPN 6 days *vs* ACEA). And the analgesic effect of ACEA on allodynia is also inhibited by AM251 (AM251+ACEA *vs* ACEA). **F, G** The duration of the ACEA-mediated analgesic effect on mechanical allodynia (9 mice). (**B–E**) The control solvent ACSF is also microinjected into the bilateral RAIC. Pain behaviors in the sham and CPN groups are not influenced by the ACSF. *P <0.05, **P <0.01, ***P <0.001, ****P <0.0001, one-way ANOVA.

Our results indicated that the EC might be involved in the modulation of the excitation and inhibition of the RAIC in the neuropathic pain state. The effect of the CB1R agonist ACEA was further explored in CPN-ligated mice (Fig. 4A). Bilateral PWTs were partially rescued to the basal level in CPN-ligated mice following bilateral microinjection of ACEA into the RAIC (CPN 6 days *vs* ACEA, bilateral *P* <0.05, Fig. 4B, C). The analgesia due to the activation of CB1Rs was alleviated by the administration of the CB1R antagonist AM251 (AM251+ACEA *vs* ACEA, bilateral *P* <0.05, Fig. 4B, C). Bilateral PWTs were not changed in CPN mice with administration of the same volume of ACSF (CPN 6 days *vs* ACSF, bilateral *P*> 0.05, Fig. 4B, C).

Similarly, the bilateral positive percentage of the innocuous stimulus markedly declined after ACEA injection into the RAIC (CPN 6 days *vs* ACEA, bilateral *P* <0.001, Fig. 4D, E). This inhibitory effect of CB1R activation on mechanical allodynia was also alleviated by AM251 (ACEA *vs* AM251+ACEA, bilateral *P* <0.05, Fig. 4D, E). ACSF, as the vehicle treatment, did not affect mechanical allodynia (CPN 6 days *vs* ACSF, bilateral *P* >0.05, Fig. 4D, E). Meanwhile, to access the duration of the analgesic role of ACEA, mechanical allodynia was measured at three time points after ACEA injection. The analgesia lasted for 25 min and declined over time (15 min, bilateral *P* <0.001; 25 min, bilateral* P* <0.05; 35 min, bilateral *P* >0.9; Fig. 4F, G).

## The Requirement of CB1Rs in RAIC GABAergic Neurons in Neuropathic Modulation

**Fig. 5:**
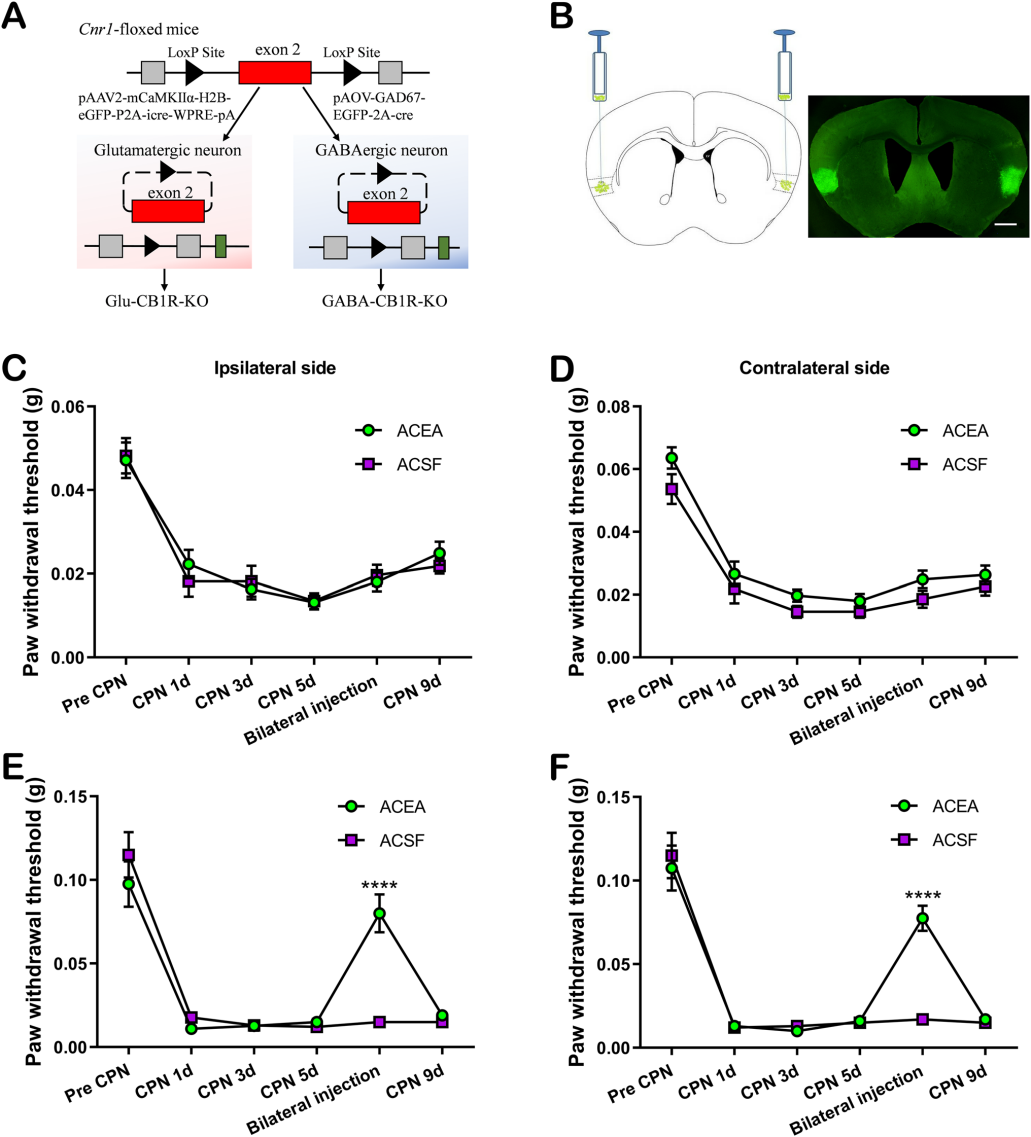
Requirement of the activation of CB1Rs in GABAergic neurons of the RAIC following CPN ligation. **A** Schematic of the structure of Cre viruses. Exon 2 of the Cnr1 gene was excised in GABAergic or glutamatergic neurons expressing Cre recombinase. **B** Left: viruses are microinjected into the bilateral RAIC of Cnr1-floxed mice according to the brain atlas. Right: representative coronal image showing the virus expression locus with green fluorescence (scale bar, 200 μm). **C, D** Conditional knockout of CB1Rs on GABAergic neurons in the RAIC. The analgesia induced by the activation of CB1Rs is blocked (bilateral injection). **E, F** Conditional knockout of CB1Rs on glutamatergic neurons in the RAIC. PWTs of bilateral CPN ligated mice increased statistically after the activation of CB1Rs (bilateral injection, ACEA, 12 mice; ACSF, 12 mice). ****P <0.0001, two-way repeated-measures ANOVA with the Bonferroni *post hoc* test.

We then asked whether the analgesic effect of CB1R activation was neuron-specific in the RAIC under the neuropathic pain state. CB1 receptors were conditionally knocked down in either glutamatergic or GABAergic neurons of the RAIC (Fig 5A, B). The conditional knockout of CB1Rs in either glutamatergic or GABAergic neurons of the RAIC did not influence the development of mechanical hyperalgesia after CPN ligation (pre-CPN *vs* CPN 1 day, all *P* <0.001, Fig. 5C–F). However, compared to wild-type mice, the bilateral baseline PWTs of Glu-CB1R^-/-^ mice but not GABA-CB1R^-/-^ mice increased significantly (Glu-CB1R^-/-^ mice, *P <*0.05; GABA-CB1R^-/-^ mice, *P >*0.05; Fig S2). Although CB1Rs were knocked down in glutamatergic or GABAergic neurons of the RAIC, bilateral PWTs were significantly decreased in CPN-ligated mice (Fig. 5C–F). The analgesic effect that referred to CB1R activation was gone following CB1R knockdown in GABAergic neurons of the RAIC in the CPN-ligated mice (at the bilateral injection point, both *P* >0.6, Fig. 5C, D), while the analgesia of CB1R activation was not changed in the CPN-ligated mice with knockdown of CB1Rs in glutamatergic neurons (at the bilateral injection point, both *P* <0.0001, Fig. 5E, F).

## Elimination of the Analgesic Effect of CB1R Activation Following DLF Lesion

**Fig. 6:**
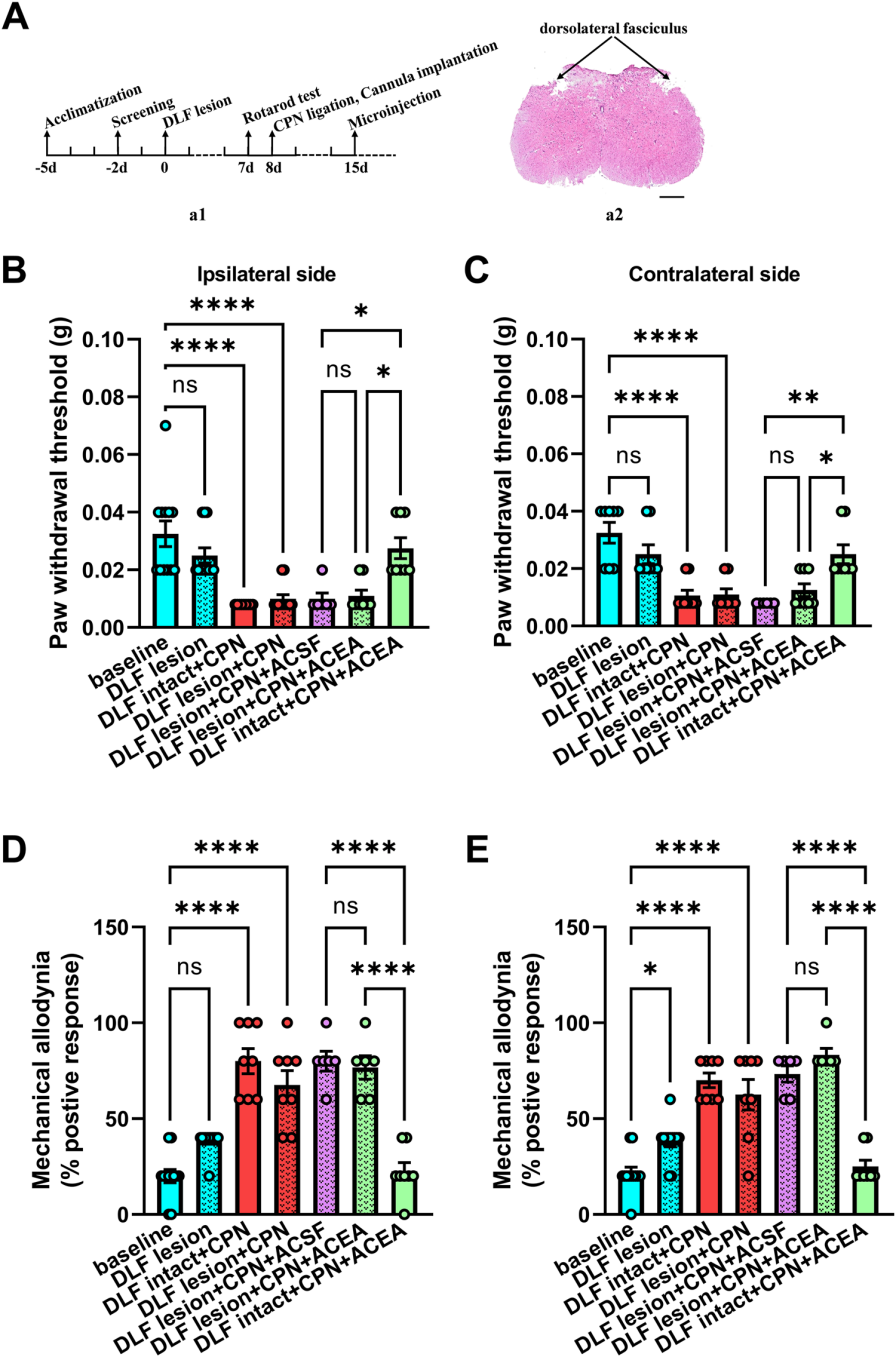
DLF lesion abolishes the analgesic effect of CB1R activation in the RAIC following CPN ligation. **A** According to the time course (**a1**), the bilateral DLF is damaged in wild mice (**a2**). Scale bar, 50 μm. Effects of a DLF lesion on both sides. The physical sensations of pain, neuropathic pain formation, and ACEA-mediated analgesia are tested by mechanical hyperalgesia (**B**, **C**) and allodynia (**D, E**). **B, C** DLF lesion does not affect the bilateral PWTs of WT mice (baseline *vs* DLF lesion). With or without a DLF lesion, the bilateral PWTs of WT mice decrease significantly after CPN ligation (baseline *vs* DLF intact+CPN and *vs* DLF lesion+CPN). The pain inhibitory effect of ACEA on mechanical hyperalgesia in CPN-ligated mice is blocked after a bilateral DLF lesion (DLF lesion+CPN+ACEA *vs* DLF intact+CPN+ACEA). **D, E** The mechanical allodynia of the contralateral but not the ipsilateral side increases significantly (baseline *vs* DLF lesion). Compared to the baseline, the mechanical allodynia increases significantly after CPN ligation (baseline *vs* DLF intact+CPN and *vs* DLF lesion+CPN). The analgesic effect of ACEA on mechanical allodynia is inhibited after a bilateral DLF lesion (DLF lesion+CPN+ACEA vs DLF intact+CPN+ACEA). Baseline, 12 mice; DLF lesion group, 12 mice; DLF intact+CPN group, 9 mice; DLF lesion+CPN group, 12 mice; DLF lesion+CPN+ACSF group, 6 mice; DLF lesion+CPN+ACEA group, 8 mice; DLF intact+CPN+ACEA group, 8 mice. *P <0.05, **P <0.01, ***P <0.001, ****P <0.0001; one-way ANOVA.

To further explore the mechanism of the analgesia due to CB1R activation in the RAIC in the neuropathic pain state, we tested the analgesic effect of CB1R activation following a DLF lesion (Fig. 6A). Compared to the baseline, bilateral PWTs were not changed after the DLF lesion (baseline *vs* DLF lesion, *P* >0.05, Fig. 6B, C). Bilateral PWTs were significantly decreased in CPN ligation groups (baseline *vs* DLF intact+CPN, and *vs* DLF lesion+CPN, all *P* <0.0001, Fig. 6B, C). Mechanical allodynia on the ipsilateral side was not changed significantly after the DLF lesion, but that of the contralateral side increased markedly (baseline *vs* DLF lesion; ipsilateral, *P* = 0.0524; contralateral, *P* = 0.0372; Fig. 6D, E).

The analgesic effect of CB1R activation on mechanical hyperalgesia in the RAIC was alleviated in CPN-ligated mice after a DLF lesion (DLF lesion+CPN+ACEA *vs* DLF intact+CPN+ACEA, bilateral *P* <0.05, Fig. 6B, C). And the analgesic effect of CB1R activation on bilateral mechanical allodynia of CPN-ligated mice disappeared following the DLF lesion (DLF lesion+CPN+ACEA *vs* DLF intact+CPN+ACEA, bilateral *P* <0.0001, Fig. 6D, E). Mechanical hyperalgesia and allodynia of CPN-ligated mice after the DLF lesion was not influenced by ACSF, which was used to treat the vehicle group (DLF lesion+CPN+ACSF *vs* DLF lesion+CPN, all *P* >0.9, Fig. 6B–E).

## Discussion

In the present study, the E/I ratio in layer V neurons was increased in the RAIC following CPN ligation. Both DSI and DSE mediated by EC signaling were induced in layer V neurons of the RAIC. DSI amplitude was significantly increased in RAIC layer V pyramidal neurons after CPN ligation, while DSE was not changed. Furthermore, the strong analgesia induced by CB1R activation in the RAIC was reversed by the CB1R antagonist AM251. The analgesia of CB1R activation required CB1Rs in GABAergic neurons of the RAIC as well following CPN ligation, but not in glutamatergic neurons. In addition, the analgesia of CB1R activation was markedly alleviated in CPN-ligated mice following a DLF lesion. These results suggested that activation of the CB1Rs on GABAergic neurons had an analgesic effect in the RAIC *via* the descending pain inhibitory pathway.

## ECs Underpin a Plastic Change in the RAIC in the Neuropathic Pain State

Changes in synaptic transmission can be influenced by the miniature postsynaptic currents [29]. Both the amplitude and frequency of mEPSCs were increased in layer II pyramidal neurons of the IC after CPN ligation [3]. Similarly, mEPSC amplitude and frequency were significantly increased in RAIC layer V pyramidal neurons after CPN ligation in our study. However, the mIPSC frequency was significantly decreased in these neurons. The E/I ratio was significantly increased in RAIC layer V pyramidal neurons after CPN ligation, suggesting that there was an imbalance of synaptic transmitters between the excitation and inhibition of these neurons in CPN-ligated mice. This result is similar to the excitatory imbalance that occurs in the IC of rats after chronic constriction injury of the sciatic nerve [5]. Neuronal activity of mPFC pyramidal neurons is increased in the early stage of neuropathic pain related to increased eCB/CB1R signaling [29]. This evidence suggests that EC-induced disturbance in the synaptic transmission in the mPFC contributes to neuropathic pain. Changes in the frequency of mEPSCs and mIPSCs indicated that presynaptic CB1Rs might be associated with an enhanced E/I ratio. It has been reported that CB1R expression is decreased in the periaqueductal gray of rats with chronic constriction injury [30], while the expression of CB1Rs in brain areas (hypothalamus, midbrain, and PFC) only increases on day 14 but not on days 3 or 7 after L5 spinal nerve ligation [31]. The expression of CB1R mRNA and protein in the IC significantly increases in rats after sciatic nerve branch ligation and transection [14]. Our results showed that the protein expression of CB1Rs in the RAIC was not changed on postoperative days 7 and 14 following CPN ligation (Fig. S3). This result is consistent with the CB1R level in the ACC in mice with inflammatory pain [12].

The EC system plays an important role in the development and persistence of neuropathic pain. DSI and DSE are two forms of synaptic plasticity that modulate cell inputs by inhibiting the excitability of presynaptic interneurons and excitatory neurons. Our results showed that both DSI and DSE exist as short-term plasticity in layer V pyramidal neurons of the RAIC. DSI amplitude significantly increased in these neurons following CPN ligation, while DSE amplitude was not changed. In contrast, the DSE amplitude of layer II/III pyramidal neurons in the ACC is reduced after nerve inflammation [12]. And there was no difference in DSI on day 7 after SNI but it decreased on day 35 [29]. Different pain models, brain regions, and experimental protocols might account for the diversity of DSI/DSE amplitude. Meanwhile, EPSCs exhibited lower sensitivity than IPSCs to a CB1R agonist [32]. It has been reported that the sensitivity of presynaptic CB1Rs also affects the variation of DSI and DSE amplitudes. Indeed, an EC-elicited decrease of inhibitory transmission facilitates the neuronal plasticity of neighboring pyramidal neurons. In the pyramidal neurons of the hippocampus, DSI-induced disinhibition initiates NMDAR-dependent long-term potentiation [33]. In the present study, an enhanced DSI indicated that the loss of GABAergic inhibition contributes to the hyperactivity of RAIC layer V pyramidal neurons.

## The Activation of CB1Rs on GABAergic Neurons has an Analgesic Effect in the RAIC *via* the Descending Pain Inhibitory Pathway

The EC system is an endogenous pain control system that plays an important role in the development and maintenance of neuropathic pain. Approaches that modulate EC metabolism and the activity of receptors influence neuropathic pain. In our study, hyperalgesia and mechanical allodynia of CPN mice were inhibited when ACEA was bilaterally microinjected into the RAIC. This result was similar to the analgesic effect of ACEA that occurs in the dorsal horn in rats with spinal nerve ligation [34]. Activation of the cannabinoid system with URB597 (an inhibitor of fatty acid amide hydrolase) in the IC inhibits mechanical allodynia in SNL rats. This analgesic effect is blocked by AM251 [14]. Our study showed that the antinociceptive effect of ACEA in the RAIC was also abolished by AM251. Thus, CB1R activation of the RAIC plays an important role in the analgesia mediated by the ECs. It has been reported that the activation of CB1Rs in GABAergic and glutamatergic neurons induce analgesia [35–37]. To identify the different roles of CB1R activation in glutamatergic or GABAergic neurons, CB1Rs were knocked out selectively in GABAergic or glutamatergic neurons of the RAIC. Mechanical hyperalgesia of RAIC GABAergic and glutamatergic CB1R^-/-^ mice was similar to wild-type mice after CPN ligation. Meanwhile, pain sensitivity is increased by whole-body CB1R knockout [38]. These results suggest that conditional ablation of CB1R in the RAIC does not affect the initiation and persistence of neuropathic pain. In our study, the analgesic effect due to the activation of CB1Rs was alleviated in CB1R-knockout in GABAergic neurons but not glutamatergic neurons. This result showed that CB1Rs of the RAIC GABAergic neurons are more important in pain modulation. The amplitude of IPSCs of RAIC layer V pyramidal neurons was decreased due to CB1R activation in the RAIC (Fig. S4). Combined with increased DSI, the activation of CB1Rs further strengthened the excitatory output of RAIC layer V pyramidal neurons. One reasonable speculation is that enhanced excitatory output of RAIC layer V pyramidal neurons has an analgesic effect associated with the descending pain inhibitory pathway.

Excitatory neurons of the RAIC project to the ventrolateral periaqueductal grey (PAG) and then activate dorsal horn neurons in the spinal cord [39]. Blockade of neuronal CB1Rs in the PAG alleviates the analgesic effects *via* electrically activating the IC [40]. Our study suggested that the activation of CB1Rs in the RAIC-produced analgesia might be related to the descending inhibitory pathway. In naïve mice, the antinociceptive effects of ACEA are absent in mice with bilateral dorsolateral funiculus damage [41]. Evidence that cannabinoid-induced analgesic effects are alleviated following a DLF lesion suggests that the analgesia might be induced by reinforcing the function of the descending pain control pathway [24]. There was no direct study referring to the effect of DLF lesions on the analgesia of CB1R activation in the RAIC. In the present study, the antinociceptive effect of activation of CB1Rs in the RAIC was attenuated after the bilateral DLF lesion. Consistent with this, the analgesic effect produced by electrical stimulation and electroacupuncture in the anterior pretectal nucleus is blocked after a DLF lesion [42, 43]. Our results confirmed that CB1R activation of the RAIC produces analgesia *via* the descending pain inhibitory pathway.

## Conclusions

**Fig. 7:**
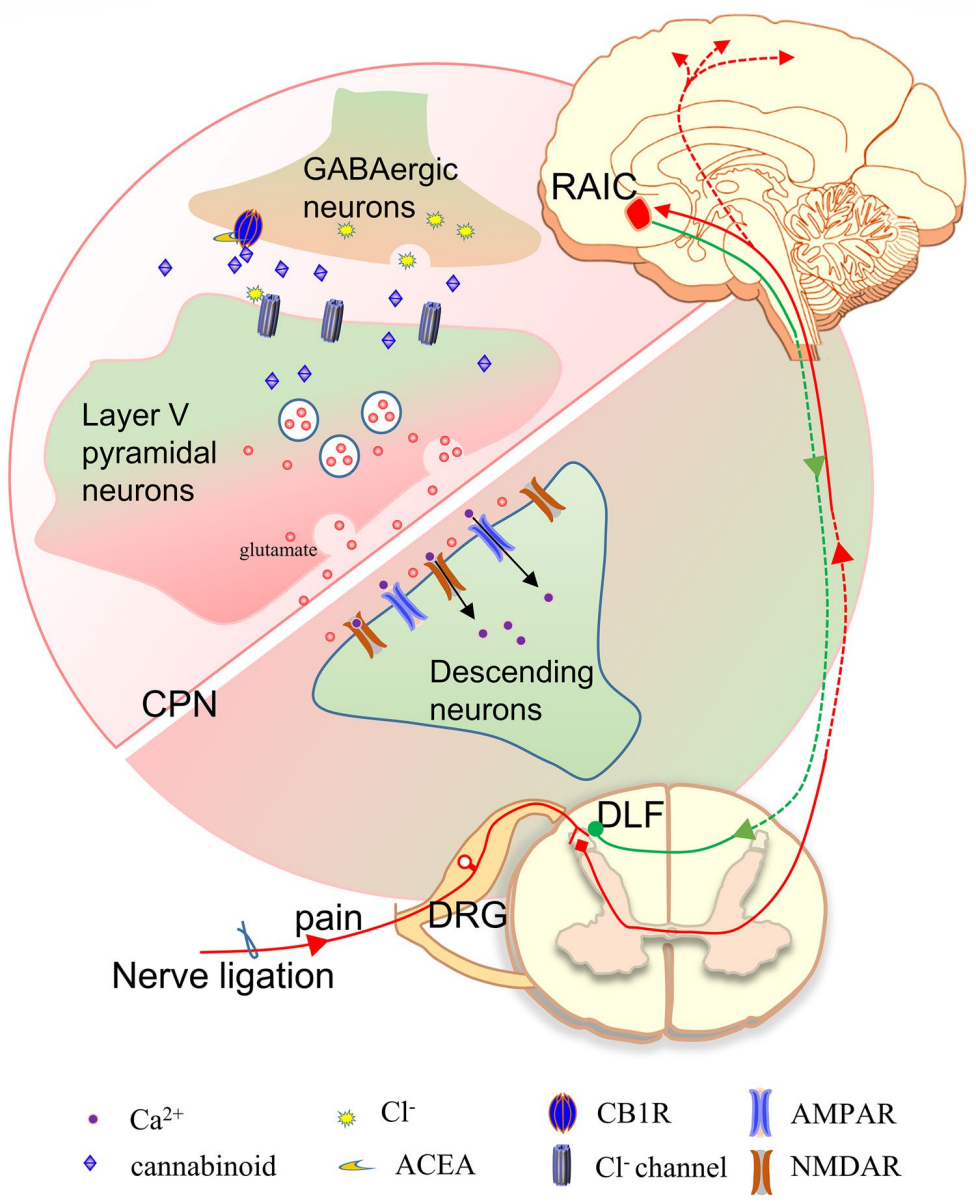
Diagram for the analgesia due to the activation of CB1Rs in GABAergic neurons through enhanced disinhibition in the RAIC. In CPN mice, CB1Rs on GABAergic neuron-mediated DSI amplitude increases. As the result, the inhibitory effect of GABAergic neurons on excitatory neurons diminishes. Then, the output of RAIC layer V pyramidal neurons increases. Finally, the action of the descending pain pathway from RAIC to DLF is enhanced. The activation of CB1Rs by ACEA (CB1R agonist) further diminishes the IPSC amplitude of RAIC layer V pyramidal neurons. Therefore, the action of the descending pain modulatory pathway is reinforced, and mechanical allodynia and hyperalgesia are blocked in CPN mice. CB1R, cannabinoid receptor 1; CPN, common peroneal nerve ligation; DLF, dorsolateral fasciculus; DRG, dorsal root ganglion; RAIC, rostral agranular insular cortex.

Excitatory synaptic transmission of RAIC layer V pyramidal neurons amplified with an enhanced E/I ratio accounts for the bilateral mechanical allodynia and hyperalgesia of mice after CPN ligation. Increased DSI but not DSE amplitude means disinhibition of interneurons on excitatory neurons. The output of RAIC layer V pyramidal neurons was enhanced after CPN ligation. The analgesic effect was lost due to the activation of CB1Rs after CB1R knockout in GABAergic neurons but not glutamatergic neurons. CB1Rs in GABAergic neurons play a more important role in the excitability of RAIC layer V pyramidal neurons in the neuropathic pain state. The CB1R activation-mediated analgesic effect in the RAIC was alleviated following a DLF lesion demonstrating that antinociception develops *via* the descending pain inhibitory pathway. In the present study, the activation of CB1Rs in RAIC GABAergic neurons (disinhibition) might produce analgesia by reinforcing the descending pain inhibitory pathway (Fig. 7). These findings provide new insight into the analgesic effect of the EC system in the central nervous system.

## Supplementary Information

Below is the link to the electronic supplementary material.

Supplementary file1 (PDF 342 KB)
