# Supplementary material for: Activation of Cannabinoid Receptor 1 in GABAergic Neurons in the Rostral Anterior Insular Cortex Contributes to the Analgesia Following Common Peroneal Nerve Ligation
Source: Neurosci Bull. 2023 Feb 11;39(9):1348–62. doi: 10.1007/s12264-023-01029-6 (PMC10465468; doi:10.1007/s12264-023-01029-6)
Supplement: Supplementary file 1 — Supplementary file1 (PDF 342 KB) [file 12264_2023_1029_MOESM1_ESM.pdf]

Supplemental Methods

Western Blot Analysis

Adult male mice (a total of 12 mice) were anesthetized with 2% isoflurane. Western blot analysis was applied as previously described [1]. 30 μg of total proteins was separated by electrophoresis on SDS-PAGE and then transferred to a polyvinylidene difluoride membrane (Millipore, USA). Membranes were incubated with primary antibody against CB1R (1:1000, Proteintech, Wuhan, China, Cat# 17978-1-AP) or β-actin (1:1000, Sigma-Aldrich, USA, Cat# A5441) overnight at 4ºC. Then the membranes were incubated with horseradish peroxidase-conjugated secondary antibodies for 1 h at room temperature. The protein bands on the membrane were visualized using an enhanced chemiluminescent kit on a Bio-Rad Image Lab system.

Supplemental Figures and Figure Legends

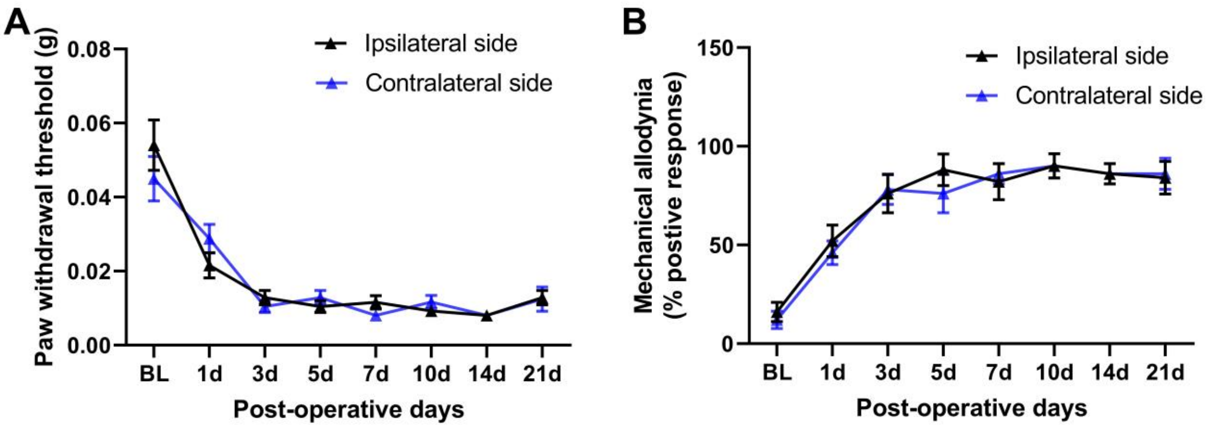

Fig. S1 No statistical difference between bilateral mechanical allodynia and hyperalgesia in CPN-ligated mice. There is no significant difference in bilateral mechanical hyperalgesia (**A**) and allodynia (**B**) of CPN-ligated mice. Differences in pain behavior between the two sides of CPN-ligated mice are tested by two-way repeated-measures analysis of variance (ANOVA) followed by the Bonferroni *post hoc* test.

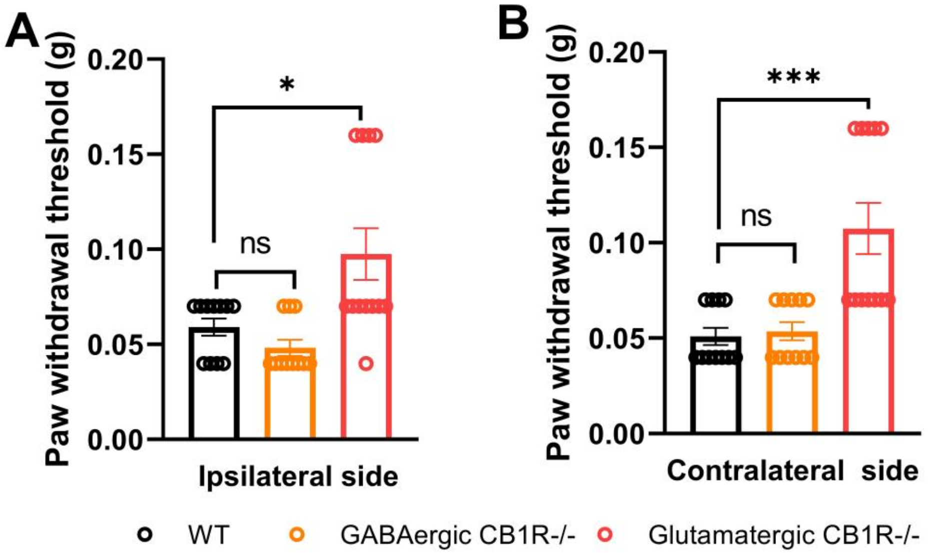

Fig. S2 The bilateral baseline of paw withdrawal thresholds is increased in the mice after the knockdown of CB1Rs in RAIC glutamatergic neurons. The ipsilateral (**A**) and contralateral side (**B**) pre-CPN paw withdrawal thresholds of Glu-CB1R$^{-/-}$ mice (n = 12) but not GABA-CB1R$^{-/-}$ mice (n = 11) are increased significantly. Compared to the PWT value for WT mice (11 mice), **P* <0.05, ****P* < 0.001, one-way ANOVA.

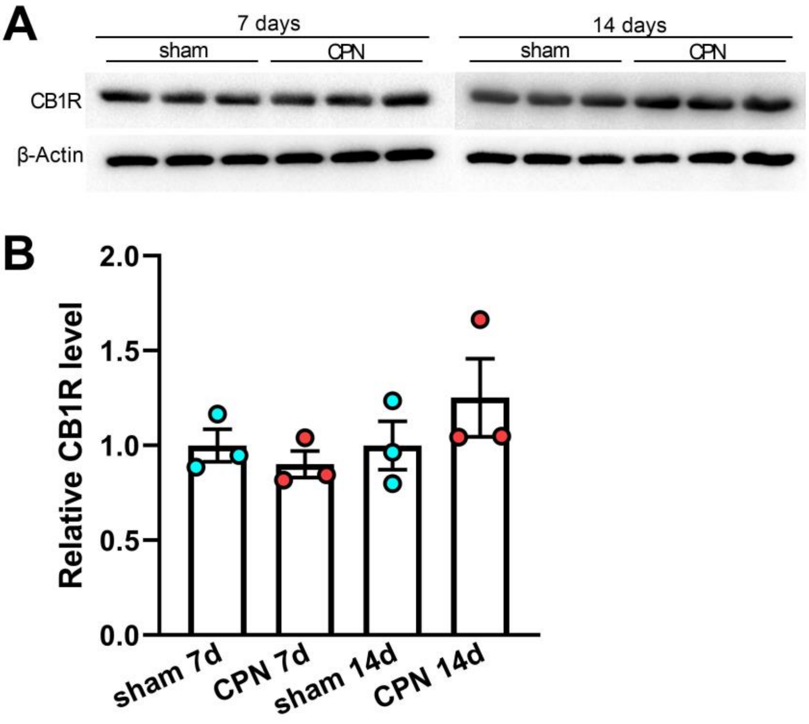

Fig. S3 The expression of CB1R does not change in the RAIC following CPN ligation. Western blots and analysis of CB1Rs in the RAIC of CPN and control groups. **A** CB1R expression and the corresponding β-actin bands at 7 days and 14 days after CPN ligation. **B** There is no significant difference in the CB1R level in the RAIC of sham and CPN-ligated mice (7 days, *P* = 0.4217, 14 days, *P* = 0.3565). *P* >0.05 *vs* sham group, *n* = 3 per group (sham group, 3 mice; CPN group, 3 mice), unpaired *t*-test.

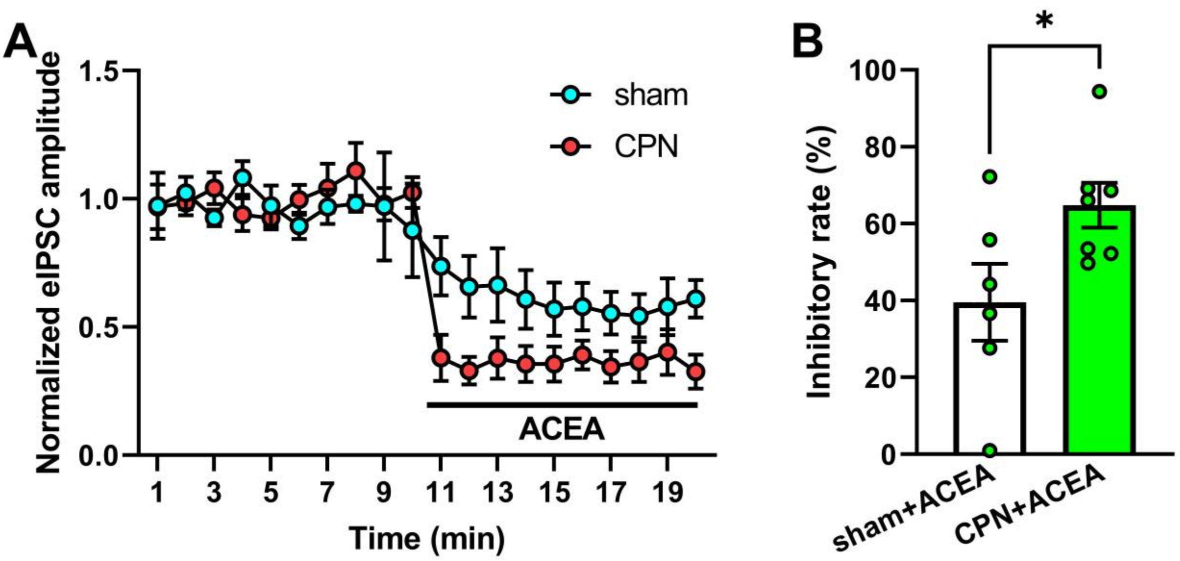

Fig. S4 ACEA decreases the amplitude of IPSCs in layer V pyramidal neurons of the RAIC following CPN ligation. **A** Amplitude of evoked IPSCs in the two groups is reduced by ACEA (2 μmol/L); **B** The inhibition of IPSC amplitude is much higher in the CPN group (*P* = 0.0448). Sham *n* = 6, CPN *n* = 7, **P* <0.05, unpaired *t*-test.

Reference:

[1] Zhao H, Xue Q, Li C, Wang Q, Han S, Zhou Y, *et al*. Upregulation of Beta4 subunit of BK_{Ca} channels in the anterior cingulate cortex contributes to mechanical allodynia associated anxiety-like behaviors. Mol Brain 2020, 13: 22.
